# Supporting the Spectrum Scholarship Program: perspectives from the Northern California and Nevada Medical Library Group

**DOI:** 10.5195/jmla.2022.1432

**Published:** 2022-04-01

**Authors:** Sarah McClung, Rachel Keiko Stark, Megan De Armond

**Affiliations:** 1 sarah.mcclung@ucsf.edu, Head of Collection Development, University of California, San Francisco, San Francisco, CA; 2 stark@csus.edu, Health Sciences Librarian, Sacramento State University, Sacramento, CA; 3 mde_armo@touro.edu, Assistant Professor and Research and Instruction Librarian, Touro University Nevada, Henderson, NV

**Keywords:** diversity, library school students, mentoring

## Abstract

The immediate past presidents and current president of the Northern California and Nevada Medical Library Group write to raise awareness of the American Library Association Spectrum Scholarship Program, share their approach to supporting Scholars in their region, and encourage Medical Library Association (MLA) chapters and MLA at large to build stronger infrastructures to support Black, Indigenous, and People of Color librarians who are in school and recently graduated.

## INTRODUCTION

As the immediate past presidents and current president of the Northern California and Nevada Medical Library Group (NCNMLG), we would like to publicly thank the Medical Library Association (MLA) for honoring our organization with the 2021 Chapter Project of the Year Award for our sponsorship, mentorship, and financial support of American Library Association (ALA) Spectrum Scholarship Program. We would like to take this opportunity to raise awareness of this scholarship program, share our approach to supporting Scholars in our region, and encourage our colleagues, MLA, and the MLA chapters to build stronger infrastructures to support Black, Indigenous, and People of Color (BIPOC) librarians who are either in school or recent graduates.

## ABOUT THE SPECTRUM SCHOLARSHIP PROGRAM

Since 1997, ALA has awarded more than 1,300 Spectrum Scholarships “to American Indian/Alaska Native, Asian, Black/African American, Hispanic/Latino, Middle Eastern and North African, and/or Native Hawaiian/Other Pacific Islander students to assist them with obtaining a graduate degree and leadership positions within the profession and ALA” [1]. Four years after ALA awarded their first Spectrum Scholarships, MLA and the National Library of Medicine (NLM) began to cosponsor Spectrum Scholarships to support students interested in health sciences librarianship [2]. The Spectrum Scholarship Program goes beyond financial assistance to address the barriers facing the recruitment and retention of BIPOC librarians by providing Scholars with leadership development and community-building resources [3]. Scholars also attend the Spectrum Leadership Institute, which provides participants professional skill–building and networking opportunities and the opportunity to attend ALA's Annual Meeting fully funded by the Institute.

## NCNMLG'S SUPPORT OF SPECTRUM SCHOLARS

In 2018, members of NCNMLG, including Spectrum Scholarship Program alumni and previous Spectrum Scholarship jurors, proposed that our chapter provide not only financial support but also professional scaffolding for a Spectrum Scholar in our region. Our executive board then unanimously voted to annually sponsor, in perpetuity, one Northern California or Nevada Spectrum Scholar interested in health sciences librarianship [4], making us the first, and currently the only, MLA chapter to provide ongoing Spectrum Scholarship funding. Our chapter intentionally chose to partner with ALA's Spectrum Scholarship Program because of its proven success supporting BIPOC library students and librarians [5]. This partnership has the added benefit of strengthening relationships between professional organizations and providing BIPOC librarians with a multifaceted support network. We have supported four Scholars to date.

In the same spirit as the Spectrum Scholarship framework [3], NCNMLG believes that financial support for formal education is just the first step toward inclusivity and social justice. To be the most effective in making our profession a more diverse one, we must also take our sponsorship further by also engaging with and mentoring the Scholars in a meaningful and sustainable way. By doing so, we believe we are helping to fill a gap that has emerged between BIPOC student support, provided through programs like the Spectrum Scholarship Program and the MLA Scholarship for Underrepresented Students [6], and leadership support, provided through programs like MLA Rising Stars [7] and ALA Emerging Leaders [8]. Success in librarianship, like in most professions, is often reliant upon who you know and having a strong and knowledgeable community to tap into. By immersing the Scholars in our regional chapter, we are providing them ready access to such a community and focused support for entry into health sciences librarianship.

There are many ways our sponsored Scholars can engage with our organization and members. In addition to encouraging their attendance and participation at our membership and executive board meetings, NCNMLG invites all Scholars to attend chapter meetings so that they can network with librarians, become familiar with professional medical library conferences, and learn about current health sciences library trends and issues. The chapter also provides the Scholars with multiple mentoring and job shadowing experiences with chapter volunteers, tailored to their professional interests and goals. Costs associated with these opportunities are covered by NCNMLG to reduce barriers to participation. We are currently accommodating Scholars remotely due to the COVID-19 pandemic and the associated library shutdowns, meeting over Zoom and encouraging virtual conference attendance. We continually adjust our support offerings in response to informal feedback from experienced librarians, current and past Spectrum Scholars, and chapter members, as well as established best practices for supporting recently graduated BIPOC librarians. We are currently developing a standardized data collection tool to document and share Scholars' voluntary feedback after they participate in our program. While this type of data collection is strategically important, we recognize we must be mindful not to place an undue burden on Scholars' time and expertise. It is important to note that not all students are interested in the extra activities, nor are all students able to participate in additional offerings beyond financial support. NCNMLG does not require students to take advantage of these engagement opportunities; they are provided to the students with encouragement.

Our chapter is proud to have recently reached two milestones regarding our support of the Spectrum Scholarship Program. First, the NCNMLG executive board unanimously voted in 2020 to create a Spectrum Scholar subcommittee to recruit more chapter members in the fundraising, mentoring, and advocacy efforts involved in fully engaging with Scholars. This development will ensure long-term, sustainable administrative assistance so that the Scholars will be provided with an abundance of support while they are in the program. Second, because we are always seeking out opportunities to raise additional funds for the program, the proceeds from the joint 2021 Mountain Pacific Health Science Libraries Conference (MPHSLC), of which NCNMLG was a collaborating chapter, were donated to the Spectrum Scholarship Program and provided funding for an additional designated MPHSLC Scholar for 2021–2022. Coordinating this donation on behalf of MPHSLC further highlighted the need to distribute the work associated with supporting the Spectrum program; relying on a single person to handle the administrative load is unsustainable and tasking a committee with these responsibilities ensures timeliness, consistency, and scalability.

## ENVISIONING THE FUTURE

Like in most other areas of librarianship, health sciences librarians are overwhelmingly white and do not accurately reflect the many populations and professions with which we frequently work. Winning the 2021 MLA Chapter Project of the Year Award has motivated NCNMLG to think even bigger about these issues of equity and inclusivity and to explore possibilities for future collaborations to fund and support more BIPOC library students who are preparing for careers in health sciences.

NCNMLG encourages other MLA chapters to engage with students who live in their region and receive an MLA/NLM-sponsored Spectrum Scholarship or the MLA Scholarship for Underrepresented Students. This support does not need to be financial in nature, although financial support is important. Offering mentorship and job shadowing opportunities, providing scholarship recipients gratis chapter membership, and including the students in chapter meetings are all ways a chapter can provide a welcoming and supportive environment. If there are chapters that already have a regional student scholarship or grant, we urge those chapters to consider also providing those students with network-building and professional opportunities in addition to financial support. An aspirational goal for NCNMLG is to be able to offer more substantive professional opportunities to Scholars, such as paid internships, and we encourage other chapters to think big when it comes to what can be accomplished through their organization.

MLA has shown continued commitment to diversity within health sciences librarianship through their longstanding financial support of the Spectrum Scholarship Program and the Scholarship for Underrepresented Students, the creation of the Diversity Task Force in 2017 [9], and the outpouring of reaffirmation of our values since the summer of 2020 in response to racist acts of violence [10]. As with the chapters, the authors would like to encourage MLA to focus its efforts on BIPOC librarians who are in school or recently graduated and consider building a stronger infrastructure to support them in their new careers. Facilitating connections between those new to MLA and their local MLA chapters, caucuses that match their goals and interests, mentoring, and funding opportunities would help meet the call for more active work from MLA on diversity, equity, and inclusion (DEI) issues. Reserving spots for BIPOC applicants in existing MLA programs, like Rising Stars and the Research Training Institute [11], will help ensure they gain the skills that will help them build their careers and connect them with a robust network of librarians. Additionally, these same MLA programs need to adequately address the unique challenges that BIPOC librarians face within the research and promotion processes to not only create supportive and inclusive environments, but to raise wider awareness of these issues in order to aid in the end of their perpetuation.

NCNMLG's support of Spectrum Scholars is just one example of what can be done, and the ideas we offer here are just some options for ways to make impactful, positive change in our organization. Overall, we want to encourage other chapters and professional groups within the MLA community to closely examine the work they are doing to support BIPOC students and librarians to make our profession a more inclusive and equitable one. We recognize that not all chapters may be able to provide financial support and professional development opportunities, but we hope that the work we have done thus far as a chapter can serve as inspiration and a roadmap for those who are able to move forward to address DEI issues in this manner. Looking to the future, NCNMLG plans to explore collaborations with other MLA chapters to sponsor additional Spectrum Scholars and to take advantage of any opportunity to expand on this successful and nationally recognized program. We will continue to incorporate feedback from the Scholars and other stakeholders to ensure that our support remains constructive and rewarding for everyone involved. A specific chapter goal is to provide assistance and collaborative opportunities for Scholars who are interested in sharing their experiences with the broader health sciences library community. We appreciate the iteration, self-reflection, and adaptation required for meaningful DEI efforts and strive to ensure that our chapter practices these critical actions. We hope that our efforts will lead to more engagement with the Spectrum Scholars within our chapter, other chapters, and MLA as a whole.
